# Prognostic value of pretreatment serum soluble interleukin-2 receptor levels in primary central nervous system lymphoma: a single-center retrospective study

**DOI:** 10.46989/001c.172867

**Published:** 2026-11-02

**Authors:** Kurumi Kondo, Toyotaka Kawamata, Nobuharu Kosugi, Masayuki Kobayashi

**Affiliations:** 1 Department of Hematology, Tokyo Metropolitan Bokutoh Hospital, Tokyo, Japan

**Keywords:** primary central nervous system lymphoma, high-dose methotrexate, soluble interleukin-2 receptor, prognosis, radiotherapy

## Abstract

Primary central nervous system lymphoma (PCNSL) is an aggressive disease with heterogeneous clinical outcomes, and readily measurable prognostic biomarkers remain limited. We retrospectively analyzed 21 patients with newly diagnosed PCNSL who received high-dose methotrexate-based therapy at a single institution between 2006 and 2023. The median age was 68 years, and 12 patients had deep-structure involvement. Median overall survival (OS) and progression-free survival were 41 and 31 months, respectively. In univariate analyses, pretreatment serum soluble interleukin-2 receptor (sIL-2R) level (p = 0.0185), cerebrospinal fluid protein level (p = 0.0401), and receipt of radiotherapy (p = 0.0377) were significantly associated with OS. Receiver operating characteristic analysis of pretreatment serum sIL-2R for 3-year OS yielded an area under the curve of 0.869; a candidate threshold of 566 U/mL showed a sensitivity of 85.7% and specificity of 83.3%. Pretreatment serum sIL-2R was associated with OS in this small retrospective cohort, and this exploratory finding warrants validation in larger independent studies.

## Introduction

Primary central nervous system lymphoma (PCNSL) is a rare and aggressive extranodal lymphoma arising in the brain, spinal cord, leptomeninges, or eyes and accounts for approximately 4% of primary central nervous system tumors.^1^ High-dose methotrexate (HD-MTX)-based chemotherapy is the backbone of treatment. Multiagent regimens such as rituximab, methotrexate, procarbazine, and vincristine (R-MPV), and consolidation with whole-brain radiotherapy (WBRT) or high-dose chemotherapy followed by autologous stem cell transplantation, are used in selected patients.^2–7^

Clinical outcomes remain heterogeneous. Established prognostic models, including the International Extranodal Lymphoma Study Group (IELSG) score and the Memorial Sloan Kettering Cancer Center (MSKCC) model, incorporate age, performance status, serum lactate dehydrogenase (LDH), cerebrospinal fluid (CSF) protein, and deep-brain involvement.^8,9^ However, additional biomarkers that are readily available in routine practice may further support prognostic assessment.

Soluble interleukin-2 receptor (sIL-2R) is widely used as an adjunctive marker in lymphoid malignancies. Although serum sIL-2R levels are often only modestly elevated in PCNSL, their association with clinical outcomes has not been well characterized.^10^ We therefore examined clinical and laboratory variables, with particular attention to pretreatment serum sIL-2R, in patients with newly diagnosed PCNSL treated at our institution.

## Materials and Methods

### Study design and patients

We conducted a retrospective single-center study of consecutive patients with newly diagnosed PCNSL who were treated between 2006 and 2023. The inclusion criteria were (i) histologically confirmed diffuse large B-cell lymphoma confined to the central nervous system and (ii) receipt of HD-MTX-based first-line therapy. Patients with systemic lymphoma or human immunodeficiency virus infection were excluded. Clinical data were obtained from electronic medical records. Laboratory variables were analyzed using available data, and missing values were not imputed. Clinical evidence of active infection or inflammation during the 30 days before treatment was assessed using medical records, body temperature, and serum C-reactive protein levels.

### Treatment

All patients received HD-MTX-based induction therapy. The recorded induction regimens were rituximab plus HD-MTX (n = 19) and R-MPV (n = 2). Five patients received at least four intrathecal injections, and 10 received radiotherapy. Consolidation and subsequent therapies were selected according to the treatment period, age, treatment response, comorbidities, functional status, physician judgment, and patient preference. The relatively limited use of radiotherapy partly reflected logistical constraints during the earlier study period, when irradiation required referral to another institution. No patient underwent autologous stem cell transplantation (ASCT), mainly because advanced age, poor performance status, or relevant comorbidities limited eligibility for intensive consolidation.

### Variables and measurements

Variables included age, gender, Eastern Cooperative Oncology Group performance status, Karnofsky Performance Status, imaging findings including deep-structure involvement, IELSG and MSKCC classifications, serum LDH, CSF cell count, CSF LDH, CSF protein, and serum sIL-2R. Pretreatment serum sIL-2R was defined as the highest value measured within 30 days before initiation of therapy. Serum sIL-2R was quantified in the hospital clinical laboratory using a chemiluminescent immunoassay with internal calibration.

### Outcomes and statistical analysis

Overall survival (OS) was defined as the time from diagnosis to death from any cause, and progression-free survival (PFS) as the time from diagnosis to progression, relapse, or death. OS and PFS were estimated using the Kaplan-Meier method, and group comparisons were performed using the log-rank test. Associations between candidate clinical or laboratory variables and OS were examined using univariate analyses. Receiver operating characteristic (ROC) analysis was used to assess the ability of pretreatment serum sIL-2R to discriminate 3-year OS and to identify a candidate cutoff. Pretreatment serum sIL-2R was additionally evaluated as a continuous variable using a univariate Cox proportional hazards model, with the hazard ratio reported per 100 U/mL increase. Exploratory Cox models including interaction terms were used to examine potential interactions of pretreatment serum sIL-2R with serum LDH and CSF protein. Because of the limited sample size and number of outcome events, multivariable analysis was not performed. All tests were two-sided, and p < 0.05 was considered statistically significant. Analyses were performed using EZR (Jichi Medical University), a graphical user interface for R.

### Ethics

This study was approved by the Institutional Review Board of Tokyo Metropolitan Bokutoh Hospital (approval no. 06-012), and was conducted in accordance with the Declaration of Helsinki. Owing to the retrospective design, the requirement for written informed consent was waived.

## Results

### Patient characteristics

Patient characteristics are summarized in **Table 1**. The median age was 68 years, and 15 of the 21 patients were male. Performance status and Karnofsky Performance Status varied widely at diagnosis. Most patients had IELSG scores of 2-3, and MSKCC classifications were predominantly classes 2 and 3. The most common initial symptoms were gait disturbance (n = 10), memory impairment (n = 5), and anorexia or general malaise (n = 5). Deep-structure involvement was observed in 12 patients. Median pretreatment serum sIL-2R and LDH levels were 445 U/mL and 235.5 U/L, respectively. Pretreatment serum sIL-2R data were available for 19 patients and serum LDH data for 20 patients. Among the 19 patients with available pretreatment serum sIL-2R data, none had fever, elevated C-reactive protein levels, or clinical evidence of active infection or inflammatory disease during the 30 days before treatment. The recorded induction regimens were rituximab plus HD-MTX (n = 19) and R-MPV (n = 2); 10 patients received radiotherapy.

**Table 1.** Patient characteristics

	Overall (N = 21)				
Age at onset, median (IQR)	68 (39.0–83.0)		PS		
Sex, Male / Female	15 / 6		0	2	
Initial presentation			1	8	
Gait disturbance	10		2	4	
Memory impairment	5		3	7	
Anorexia / General malaise	5		KPS		
Aphasia / Dysarthria	3		90	6	
Fatigue	3		80	2	
Impaired consciousness	2		70	3	
Tumor location			60	1	
Lateral ventricles	6		50	5	
Parietal lobe	5		40	4	
Basal ganglia / thalamus	5		IELSG score		
Cerebellum	3		0–1	4	
Frontal lobe	3		2–3	11	
Temporal lobe	2		4–5	6	
Parietal convexity	2		MSKCC score		
Treatment			Class 1	3	
R + HD-MTX	19		Class 2	9	
RMPV	2		Class 3	9	
+ Intrathecal injection ≥4 times	5		sIL-2R, median (IQR)	445 (122–1414)	
+ Radiation	10		Serum LDH, median (IQR)	235.5 (166–526)	

PS, performance status; KPS, Karnofsky Performance Status; IELSG, International Extranodal Lymphoma Study Group; MSKCC, Memorial Sloan Kettering Cancer Center; HD-MTX, high-dose methotrexate; R, rituximab; RMPV, rituximab, methotrexate, procarbazine, and vincristine.

### Survival outcomes

The median OS was 41 months (**Figure 1A**), and the median PFS was 31 months (**Figure 1B**). The survival curves showed substantial heterogeneity, with several early events and a smaller group of long-term survivors.

**Figure 1.**
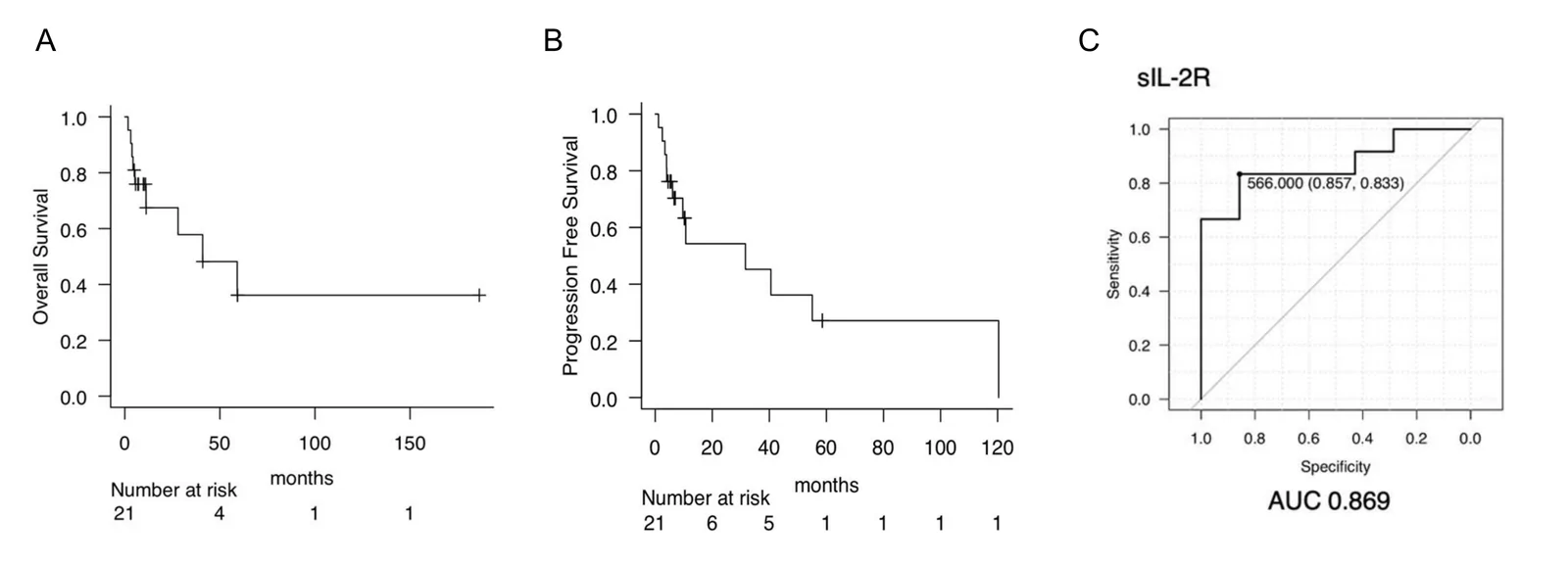
Survival outcomes and ROC analysis. **(A)** Overall survival of patients with primary central nervous system lymphoma. Overall survival was estimated using the Kaplan-Meier method. Tick marks indicate censored observations, and numbers at risk are shown below the x-axis. **(B)** Progression-free survival of patients with primary central nervous system lymphoma. Progression-free survival was estimated using the Kaplan-Meier method. Tick marks indicate censored observations, and numbers at risk are shown below the x-axis. **(C)** Receiver operating characteristic curve of pretreatment serum soluble interleukin-2 receptor for 3-year overall survival. The area under the curve was 0.869. The plotted point corresponds to a candidate threshold of 566 U/mL, with a sensitivity of 85.7% and specificity of 83.3%.

### Prognostic analyses

Associations between clinical and laboratory variables and OS are shown in **Table 2**. Pretreatment serum sIL-2R level (p = 0.0185), CSF protein level (p = 0.0401), and receipt of radiotherapy (p = 0.0377) were significantly associated with OS in univariate analyses. Analysis of pretreatment serum sIL-2R as a continuous variable using univariate Cox regression also showed an association with shorter OS (HR 1.80 per 100 U/mL increase, 95% CI 1.24–2.61; p = 0.0022). Serum LDH showed a trend toward association with OS (p = 0.0834). ROC analysis of pretreatment serum sIL-2R for 3-year OS yielded an area under the curve of 0.869 (**Figure 1C**). A candidate threshold of 566 U/mL showed a sensitivity of 85.7% and specificity of 83.3%. Exploratory Cox proportional hazards analyses showed no significant interaction of pretreatment serum sIL-2R with serum LDH (p for interaction = 0.740) or CSF protein (p for interaction = 0.699) with respect to OS.

**Table 2.** Univariate analysis of prognostic factors

Variables	Category	N (%)	P value
Age of onset (median)	<70	13 (61.9)	0.118
	≥70	8 (38.1)	
Sex	Male	15 (71.4)	0.225
	Female	6 (28.6)	
PS	0	2 (10)	0.825
	1	8 (38)	
	2	4 (14)	
	3	7 (33)	
KPS	90	6 (29)	0.567
	80	2 (10)	
	70	3 (14)	
	60	1 (5)	
	50	5 (24)	
	40	4 (19)	
IELSG score	0–1	4 (19.0)	0.336
	2–3	11 (52.4)	
	4–5	6 (28.6)	
MSKCC score	class 1	3 (14.3)	0.361
	class 2	9 (42.9)	
	class 3	9 (42.9)	
Treatment	Intrathecal injection ≥4 times	5 (23.8)	0.712
	Radiation+	10 (47.6)	0.0377
Deep lesion	Presence	12 (57.1)	0.409
	Absence	9 (42.9)	
Serum LDH (U/L)	<220	9 (42.8)	0.0834
	≥220	11 (52.4)	
sIL-2R (U/mL)	<450	9 (42.8)	0.0185
	≥450	10 (47.6)	
CSF cell count (cells/μL)	<20	7 (33.3)	0.184
	≥20	5 (23.8)	
CSF LDH (U/L)	<40	4 (19.0)	0.128
	≥40	4 (19.0)	
CSF protein (mg/dL)	<100	4 (19.0)	0.0401
	≥100	8 (38.0)	
Days from symptom onset	<25	10 (47.6)	0.408
to diagnosis	≥25	11 (52.4)	
Days from symptom onset	<50	10 (47.6)	0.119
to treatment	≥50	11 (52.4)	

Note: The Log-rank test was used for survival analysis.

## Discussion

In this retrospective single-center study of 21 patients with PCNSL treated with HD-MTX-based therapy, pretreatment serum sIL-2R was associated with OS in univariate analysis. The observed median OS of 41 months and PFS of 31 months were within the range reported in previous HD-MTX-based cohorts.^11–13^ These findings support further evaluation of serum sIL-2R as a potential prognostic biomarker in PCNSL. However, given the small sample size and absence of multivariable analysis, the present findings should be regarded as exploratory and do not establish sIL-2R as an independent prognostic factor.

sIL-2R reflects lymphocyte activation and tumor burden in several lymphoid malignancies. Serum levels in PCNSL are often modest, possibly because the blood-brain barrier limits the movement of tumor-associated proteins into the systemic circulation.^10^ Even within this relatively low range, higher pretreatment serum sIL-2R levels were associated with inferior survival. The ROC analysis also showed good discriminatory performance for 3-year OS, supporting the potential value of sIL-2R as a simple biomarker available in routine clinical practice.

The IELSG and MSKCC models are based mainly on age, performance status, LDH, CSF protein, and disease distribution.^8,9^ In the present cohort, CSF protein was also associated with OS, whereas serum LDH showed a nonsignificant trend. Because multivariable analysis was not feasible, whether serum sIL-2R provides prognostic information independent of or incremental to these established models remains uncertain. If validated in larger cohorts, serum sIL-2R could potentially complement established prognostic models in baseline risk assessment and prognostic counseling. Its role in guiding treatment or follow-up cannot be determined from the present study. The data-derived cutoff of 566 U/mL should be regarded as exploratory and requires validation in an independent cohort.

Receipt of radiotherapy was also associated with longer OS. This finding should be interpreted in the context of treatment selection, because patients who received radiotherapy may have differed in age, functional status, treatment response, or other clinical characteristics. In addition, the study period spanned 2006-2023, and treatment strategies evolved over time. Only two patients received R-MPV, and the cohort was therefore not fully representative of current standardized treatment approaches. Nevertheless, the heterogeneous real-world cohort reflects the clinical spectrum encountered in routine practice.

This study has several limitations. The retrospective single-center design and small sample size limited statistical power and precluded reliable multivariable analysis. Treatment strategies were heterogeneous, and some laboratory variables were unavailable in a subset of patients. Serum sIL-2R is a nonspecific biomarker and may be influenced by conditions other than lymphoma. However, none of the 19 evaluable patients had fever, elevated C-reactive protein levels, or clinical evidence of active infection or inflammatory disease during the pretreatment period. Therefore, acute infection or inflammation was unlikely to account for the observed association between serum sIL-2R and OS. Nevertheless, possible effects of other factors, including renal function and comorbidities, cannot be completely excluded. In addition, the candidate ROC cutoff was derived and evaluated in the same small cohort and may therefore be subject to overfitting and optimistic performance estimates; external validation is required. Despite these limitations, the consistent association between serum sIL-2R and OS supports further evaluation of this readily available biomarker in larger multicenter studies.

## Conclusion

Pretreatment serum sIL-2R was significantly associated with OS in patients with PCNSL and showed good discriminatory performance for 3-year survival. Larger independent studies are warranted to determine its prognostic value beyond established clinical factors.

### Author contributions

Kurumi Kondo: investigation, data curation, and writing - original draft. Toyotaka Kawamata: investigation and writing - review and editing. Nobuharu Kosugi: investigation and writing - review and editing. Masayuki Kobayashi: conceptualization, methodology, supervision, formal analysis, and writing - review and editing. All authors reviewed and approved the final manuscript.

### Declaration of competing interest

The authors declare no conflicts of interests.
